# Biological Characteristics of the Cytochrome P 450 Family and the Mechanism of Terpinolene Metabolism in *Hyalomma asiaticum* (Acari: Ixodidae)

**DOI:** 10.3390/ijms252111467

**Published:** 2024-10-25

**Authors:** Caishan Li, Xueqing Zhao, Wenlong Liu, Licui Wen, Yuqian Deng, Wenyu Shi, Na Zhou, Ruiqi Song, Ercha Hu, Qingyong Guo, Bayinchahan Gailike

**Affiliations:** 1 College of Veterinary Medicine, Xinjiang Agricultural University, Urumqi 830052, China; caishanlivet@outlook.com (C.L.); 320232820@stu.xjau.edu.cn (X.Z.); 320240083@stu.xjau.edu.cn (L.W.); 320232830@stu.xjau.edu.cn (Y.D.); 320222767@stu.xjau.edu.cn (W.S.); 320232901@stu.xjau.edu.cn (N.Z.); huercha@xjau.edu.cn (E.H.); 2 Xinjiang Key Laboratory of New Drug Study and Creation for Herbivorous Animals, Urumqi 830052, China; 3 School of Chemistry and Chemical Engineering, Guangxi University, Nanning 530004, China; wilen886@st.gxu.edu.cn; 4 School of Medicine, Shihezi University, Shihezi 832003, China; songruiqi@shzu.edu.cn; 5 Veterinary Medicine Postdoctoral Research Station of Xinjiang Agricultural University, Urumqi 830052, China

**Keywords:** CYP450, gene family, monoterpenoid, genome, transcriptome, molecular docking

## Abstract

The CYP450 enzyme is a superfamily enzyme ubiquitously found in nearly all organisms, playing a vital role in the metabolism of both endogenous and exogenous compounds, and in biosynthesis. Unfortunately, an understanding of its classification, functions, expression characteristics, and other biological traits in *Hyalomma asiaticum*, a vector for Crimean–Congo Hemorrhagic Fever, as well as of the genes implicated in its natural product metabolism, is lacking. Towards this end, this study has identified 120 *H. asiaticum* CYP450 genes via transcriptome data in the face of a joint genome threat from terpinolene. The proteins these genes encode are of higher molecular weight, devoid of a signal peptide, and composed of unstable hydrophobic proteins principally containing 1–3 variable transmembrane regions. Phylogenetic evolution classifies these *H. asiaticum* CYP450 genes into four subfamilies. These genes all encompass complete CYP450 conserved domains, and five specific conserved motifs, albeit with different expression levels. GO and KEGG annotation findings suggest a widespread distribution of these CYP450 genes in many physiological systems, predominantly facilitating lipid metabolism, terpenoid compound metabolism, and polyketone compound metabolism, as well as cofactor and vitamin metabolism at a cellular level. Molecular docking results reveal a hydrophobic interaction between the ARG-103, ARG-104, LEU-106, PHE-109, and ILE-119 amino acid residues in CYP3A8, which is primarily expressed in the fat body, and terpinolene, with a notably up-regulated expression, with affinity = −5.6 kcal/mol. The conservation of these five key amino acid residues varies across 12 tick species, implying differences in terpinolene metabolism efficacy among various tick species. This study thereby fills an existing knowledge gap regarding the biological characteristics of *H. asiaticum* CYP450 genes and paves the way for further research into the functions of these particular genes.

## 1. Introduction

Ticks serve as biological vectors for numerous pathogens, with their species possibly expanding in response to clinical and environmental changes [1]. For a considerable period, chemical acaricides have been the primary method utilized for tick and mite control [2]. At the same time, the environmental pollution caused by the use of chemical acaricides, as well as the contamination of meat and milk, is of equal concern [3].

The prolonged application of chemical acaricides has resulted in the issue of tick resistance [4]. One form of this resistance stems from metabolic adjustments facilitated by detoxification enzymes [5]. CYP450 is distributed in all organisms, including animals, plants, and fungi, and has a remarkable catalytic ability to promote more than 20 types of oxido-reactions, including oxidation and epoxidation [6,7]. Cytochrome p 450 (CYP 450) is the phase Ⅰ detoxification enzyme of the insect detoxification system [8]. In traditional classification, insect CYP450s have five conserved motifs (Helix–C, Helix–I, Helix–K, PERF, and heme-binding), which have been classified as mitochondrial CYP clan, CYP2 clan, CYP3 clan, and CYP4 clan [9]. Nevertheless, recent reclassifications have introduced two additional categories, the CYP20 and CYP16 clans [10]. CYP450s are integral to numerous insect life processes. More specifically, CYP2 is involved in ecdysteroid and juvenile hormone biosynthesis [10,11], while mitochondrial CYPs participate in the metabolism of fatty acids, sterols, and hormones. The CYP3 gene plays a role in the synthesis of insecticides (CYP6 and CYP9 families in the CYP3 clan [12]) and the metabolism of plant secondary metabolites. CYP4 is linked to pheromone metabolism [12,13]; however, the functions of CYP20 and CYP16 remain uncertain.

Extensive studies at the molecular level have investigated the role of CYP450s in chemical acaricide resistance. For example, CYP389C16 has been identified as a factor in the cross-resistance of *Tetranychus cinnabarinus* to cyflumetofen and pyridaben [14]. Similarly, CYP4CL2 in *Panonychus citri* has proved to be associated with resistance to pyridaben metabolism [15]. In *Tetranychus cinnabarinus*, CYP389B1 and CYP392A26 show association with fenpropathrin resistance [16]. Moreover, in ticks, increased CYP450 enzyme activity is directly linked to pyrethroid resistance in *Rhipicephalus* (*Boophilus*) *annulatus* [17]. Furthermore, the heightened expression of CYP41 and CYP3006G8 in *Rhipicephalus microplus* has been found to be associated with pyrethroid resistance [18]. Beyond its connection to chemical acaricide resistance, CYP450s are also linked with the metabolism of plant essential oils or natural products. For instance, CYP450s in *Sitophilus zeamais* are implicated in the metabolism of the natural product terpinen–4–ol [19]. Also, CYP6BQ7 in *Tribolium castaneum* shows metabolic relevance to *Artemisia vulgaris* essential oil [20]. Additionally, CYP6CR2 and CYP6DE5 in *Dendroctonus armandi* are involved in the metabolism of allelochemicals in host plants [21]. Moustafa MAM’s molecular docking investigation has displayed that *Spodoptera littoralis* CYP101C1 is involved with the metabolizing of citrus EO and citral [22].

Given the confluence of risks posed by chemical acaricides in tick control, plant extracts or natural products represent novel and potent alternatives to chemical acaricides [23,24]. There has been considerable evaluation of the efficacy of various plant essential oils and natural products in tick control [25,26,27]. Terpinolene, a monoterpene derived from plant essential oils [28,29], has demonstrated noteworthy repellent effects against *Dermacentor Variabilis* [30]. Additionally, it has been effective in eradicating blood-sucking arthropod mosquito larvae [31,32]. Terpinolene’s dual function as a killing and repellent agent positions it as a promising prospect for the formulation of natural, effective tick control products.

To date, relatively little is known about the molecular mechanisms by which effective natural products contribute to tick control. Considering that members of the CYP450 family in other species have been shown to play key roles in metabolizing chemical pesticides or natural products, we hence hypothesize that members of the CYP450 gene family in *Hyalomma asiaticum* (a biological vector of Crimean–Congo Hemorrhagic Fever Virus [33]) are also crucial in the mechanism of metabolizing natural products.

This study marks the first systematic analysis of the genetic evolution, expression characteristics, and function of the CYP450 gene family in *H. asiaticum*, based on genome-associated, qRT-PCR-validated transcriptomic data. Using the molecular docking method, we also explored the binding mode of terpinolene to the candidate CYP450 genes in *H. asiaticum*, which metabolize it. These findings will provide a theoretical foundation for further exploration of the tick’s CYP450-mediated monoterpene metabolism mechanism during tick control. The results offer fresh insights into the function of the tick CYP450 gene family at the molecular level, having significant scientific and practical implications on tick control research.

## 2. Results

### 2.1. Nymphicidal Activity

The results from the NIT experiment demonstrated that the LC_50_ of terpinolene against unfed nymph *H. asiaticum* was 6.89 mg/mL (95% CI = 6.08–7.83 mg/mL), and the regression equation was represented as *y* = 0.0514*x* + 0.023 (*R*² = 0.96). The difference analysis results showed that the difference between the two different concentration groups was extremely highly significant (*p* < 0.01) (Figure S1 in File S1).

### 2.2. Effect of Terpinolene on the MFO Enzyme Activity of H. Asiaticum

Relative to MFO enzyme activities in the control group, MFO activities were up-regulated after both LC_20_ and LC_50_ treatments, and slightly down-regulated after LC_80_ treatment, but still higher than in the control group. Significant difference analysis showed that MFO enzyme activities are extremely significant between the control group and LC_50_ (*p* < 0.01) (Figure S2 in File S1).

### 2.3. Transcriptome Data Overview

The transcriptome data related to genome association were derived from five samples each from the control and treatment groups. After removing the adapter, low-quality reads, polyA, and N (%), clean data as a percentage of raw reads exceeded 99% for each sample in both the control and treatment groups, with a Q20 value standing at approximately 97–98% for clean data (Figure S3 in File S1). Additionally, the GC content in *H. asiaticum* contigs was about 48–50% (Table S1 in File S1). The clean reads that did not match the number of reads in the ribosome stood at around 95–99% (Table S2 in File S1). There was about an 87–88% similarity in the number of reads that could all be localized to the genome and the percentage of valid reads, while reads with multiple and unique comparisons to the reference genome amounted to about 2% and 85–86% of valid reads, respectively (Table S3 in File S1). The comparative results between the reference region of the reads with localized sample species and the genome was grouped into exons (around 52–55%), introns (about 9–10%), and spacer regions (approximately 36–39%) (Figure S4 in File S1). A total of 5361 new genes were identified, with the total number of genes detected by sequencing standing at over 46–47% of all genes (Table S4 in File S1). The qRT-PCR expression trends of the six differentially expressed genes were consistent, revealing insignificant differences from the RNA-seq data (*p* > 0.05), implying the transcriptome expression data were reliable (Figure S5 in File S1).

### 2.4. Physicochemical Properties of Genome-Wide CYP450 Genes in H. Asiaticum

A total of 120 CYP450 genes were extracted from the *H. asiaticum* genome (File S2), including 35 newly identified genes (prefixed with MSTRG). Three genes (KAH6930275.1, KAH6930969.1, and MSTRG.28517) were segmented from a single gene into two as a part of gene correction. These genes encode proteins ranging in size from 361 (KAH6926449.1) to 1098 (MSTRG.15397, a novel gene) amino acids. Physicochemical property analysis reveals that the molecular weights of these proteins vary from 41.51 to 122.48 kDa. Among them, 107 proteins with an isoelectric point (*p*I) greater than 7 are basic amino acids; the rest are acidic. Eighty unstable proteins have an instability index greater than 40, whereas the rest are stable. A hundred and eighteen proteins have a negative hydrophobicity index and are hence hydrophilic, leaving the other two proteins to be hydrophobic. Signal peptide prediction shows that none of the 120 CYP450 genes from *H. asiaticum* contain signal peptides. According to transmembrane region analysis, forty-nine proteins have no transmembrane regions, fifty-five have one, fifteen have two, and one protein (KAH6926453.1) has three (Table S1).

### 2.5. Phylogenetic Analysis and Expression Pattern of CYP450 in H. Asiaticum

**Figure 1:**
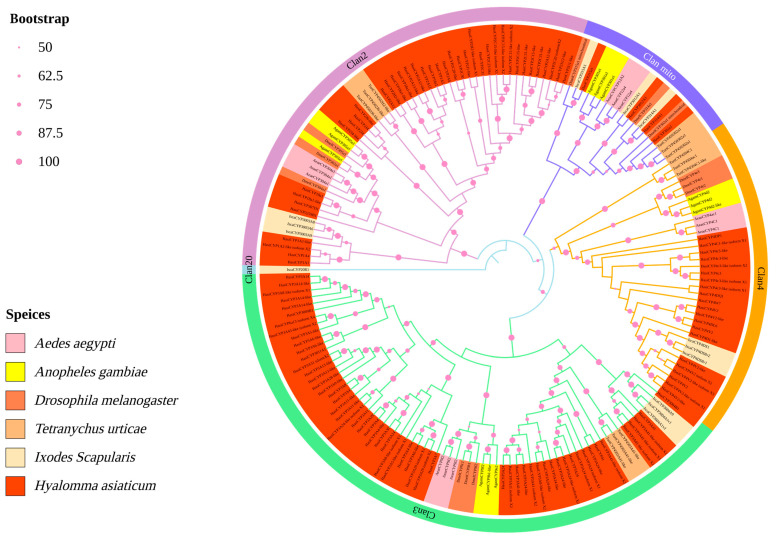
The analysis of the genetic evolution of CYP450 in *H. asiaticum*.

**Figure 2:**
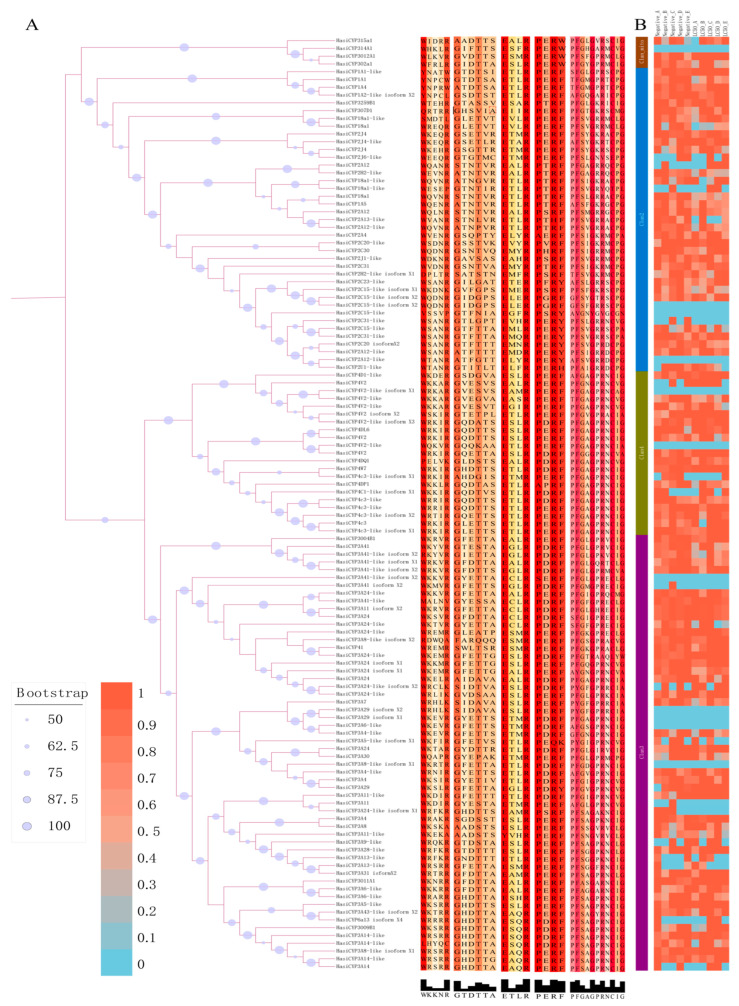
The distribution and expression patterns of conserved motifs within the CYP450 family of *H. asiaticum.* (**A**) Analysis of conserved motifs of CYP450 family members in *H. asiaticum*. (**B**) Expression profiles of CYP450 family members in *H. asiaticum* under terpinolene stress.

In order to examine the relationships among CYP450s of *H. asiaticum* and various species, we constructed a maximum likelihood genetic tree. This genetic tree classified the CYP450s of *H. asiaticum* and other species into clan2, clan3, clan4, and clan mito (Figure 1). The homology between CYP450s of *H. asiaticum* and those of *Ixodes scapularis* is the most pronounced. However, each clan contains a different number of CYP450s, and from highest to lowest, they are as follows: clan3 (56), clan2 (39), clan4 (21), and clan mito (4), with no presence in clan20 (Figure 1). By analyzing conserved motifs in 120 *H. asiaticum* with complete CYP450 structural domains, we discovered that all included the Helix–C, Helix–I, Helix–K, PERF, and heme-binding motifs (Figure 2A). We constructed an expression heatmap based on TPM values to examine the expression pattern of different CYP450 genes in *H. asiaticum* (Figure 2B). Interestingly, not all *H. asiaticum* CYP450 genes show the same expression patterns. Specifically, nine, forty-one, and seventy genes showed high, medium, and low expression levels, respectively. The highly expressed genes were found in clan3 (7) and clan2 (2). Moderately expressed genes were found in clan3 (22), clan2 (14), and clan4 (5). No clan mito genes were represented among either the highly or moderately expressed genes (Table S2).

### 2.6. Functional Annotation of the CYP450 Genes in H. Asiaticum

**Figure 3:**
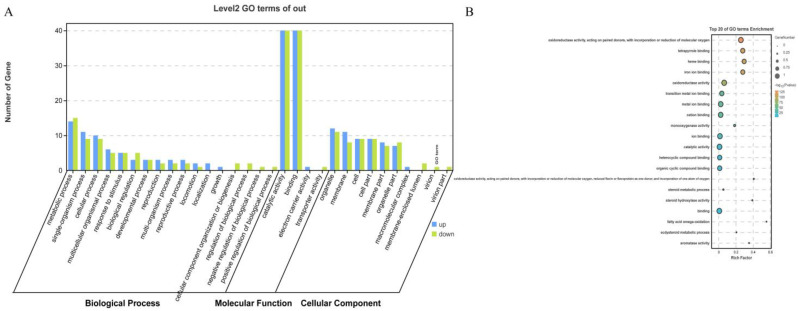
Functional annotation of the CYP450 gene family within the GO in *H. asiaticum* under terpinolene stress. (**A**) Functional analysis of CYP450 family GO terms in *H. asiaticum*. (**B**) Enrichment analysis of CYP450 family GO terms in *H. asiaticum*.

The gene function of CYP450 in *H. asiaticum* was further analyzed using GO enrichment analysis and KEGG pathway annotation. The results of GO functional annotation showed that the CYP450 gene in *H. asiaticum* had 130, 162, and 113 annotations for biological processes, molecular functions, and cellular components, respectively, and were classified into 31 level2 categories. Members of the secondary categories were categorized into biological processes (17), cellular components (10), and molecular functions (4) based on the number of GO entries they contained. Biological processes mainly include metabolic processes (GO: 0008152), single-organism processes (GO: 0044699), and cellular processes (GO: 0009987). Among them, three genes (KAH6930275.1, KAH6930993.1, and KAH6933706.1) were up-regulated in the detoxification process (GO: 0098754). The cellular components mainly consisted of organelle (GO: 0043226), cell membrane (GO: 0016020), and cell (GO: 0005623). The molecular functions are primarily related to catalytic activity (GO: 0003824), binding (GO: 0005488), etc. (Figure 3A). CYP450 genes are primarily enriched in the level2 classifications under two of the molecular functions entries, these being catalytic activity and binding, which contain the highest number of genes (80 genes included in each, 40 each in up- and down-regulated); metabolic processes within the biological processes category contain the highest number of genes, this being 29 (up-regulated: 14, down-regulated: 15); and organelles in cellular components contain the highest number of genes, at 23 (up-regulated: 12, down-regulated: 11) (Figure 3A). Oxidoreductase activity, tetrapyrrole binding, and heme binding were sequentially enriched in the top three of the twenty GO-enriched entries (Figure 3B).

**Figure 4:**
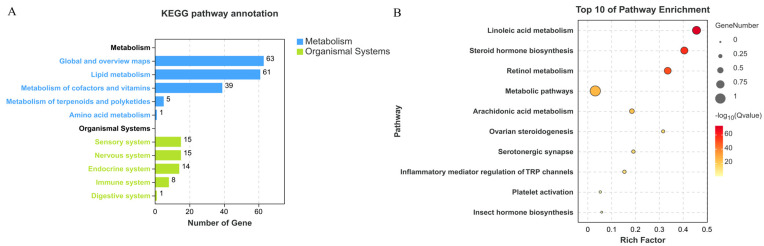
Analysis of the KEGG pathway of CYP450 gene family in *H. asiaticum* under terpinolene stress. (**A**) Annotation of the KEGG pathway of the CYP450 family in *H. asiaticum*. (**B**) Enrichment of the KEGG pathway of the CYP450 family in *H. asiaticum*.

KEGG pathway annotation annotates the CYP450 gene of *H. asiaticum* according to metabolic and organismal systems. The metabolism category includes global and overview maps, lipid metabolism, metabolism of cofactors and vitamins, and metabolism of terpenoids and polyketides. Lipid metabolism and global and overview maps were the metabolic classes containing the most genes, with values of 61 and 63, respectively (Figure 4A). Pathway enrichment results showed that the top 10 pathways involved in CYP450 were, in order, linoleic acid metabolism (ko00591), steroid hormone biosynthesis (ko00140), retinol metabolism (ko00830), metabolic pathways (ko01100), arachidonic acid metabolism (ko00590), ovarian steroidogenesis (ko04913), serotonergic synapses (ko04726), inflammatory mediator regulation of TRP channels (ko04750), platelet activation (ko04611), and insect hormone biosynthesis (ko00981) (Figure 4B).

### 2.7. Screening and Tissue Distribution of CYP450 Genes in H. Asiaticum Involved in Terpinolene Metabolism

**Figure 5:**
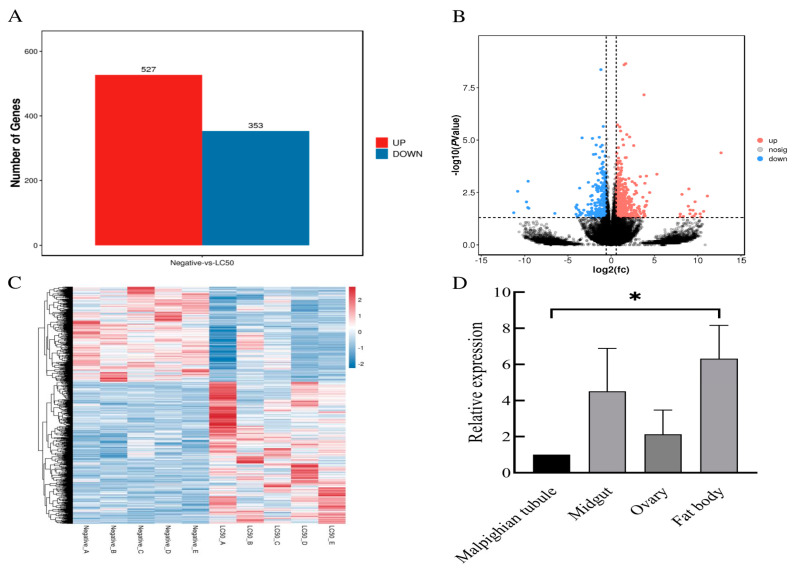
Genes differentially expressed under terpinolene stress: (**A**) statistics pertaining to the count of differentially expressed genes; (**B**) the volcano plot representation of differentially expressed genes; (**C**) a heatmap illustrating hierarchical clustering of differentially expressed genes; (**D**) results showing the relative expression of significantly up-regulated *H. asiaticum* CYP3A8 genes within varying tissues. Note: * indicates significant differences (*p* < 0.05) in CYP3A8 gene between malpighian tubule and fat body.

In the transcriptome under terpinolene stress, a total of 880 distinctly differentially expressed genes were detected. Of course, 527 were notably up-regulated, while 353 were significantly down-regulated (Figure 5A). The distribution of these differentially expressed genes is represented in a volcano diagram (Figure 5B); the genes closer to the diagram’s ends exhibited a greater degree of difference. A hierarchical clustering of differential gene expression patterns was performed, and the clustering results were presented using heatmaps (Figure 5C). It is plausible that these genes with similar expression patterns share standard functions or engage in similar metabolic or signaling pathways. The hierarchical clustering of the differentially expressed genes between the control and treatment groups is clearly displayed (Figure 5C). CYP3A8 (KAH6930993.1, Gene Bank accession: PQ058615), one of the differentially expressed genes, showed substantial up-regulation and high expression; we hypothesize that it plays a critical role in terpinolene metabolism. The distribution of CYP3A8 gene expression was further examined in the malpighian tubule, midgut, ovary, and fat body tissues, using CYP3A8 gene expression in the malpighian tubule as a point of reference. The findings revealed CYP3A8 expression in all examined tissues, with elevated expression in the midgut and the fat body. Significant disparities (*p* < 0.05) in CYP3A8 expression in *H. asiaticum* were observed between the malpighian tubule and the fat body (Figure 5D).

### 2.8. Mechanism of Terpinolene Metabolism by the CYP450 Gene of H. Asiaticum

**Figure 6:**
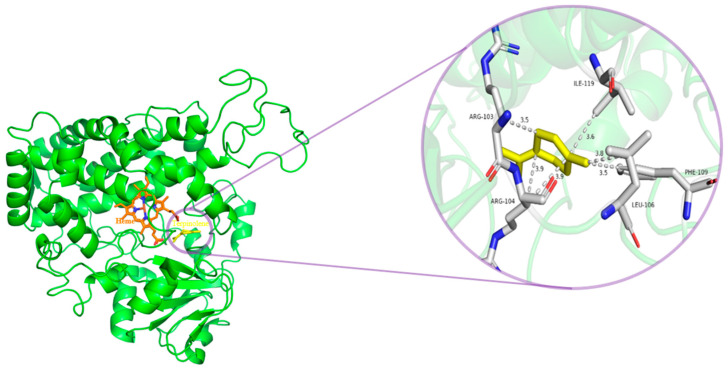
The results of molecular docking between terpinolene and CYP3A8 of *H. asiaticum*.

This study seeks to extensively understand the mechanism of action of the *H. asiaticum* CYP450 gene, specifically its role in the metabolism of terpinolene and the conserved of critical amino acid residues. Initially, we examined the binding mode of the protein encoded by the CYP3A8 gene—found in *H. asiaticum*—which exhibits high expression in the terpinolene stress transcriptome (receptor), and evaluated its interaction with terpinolene (ligand) through molecular docking. Following this, we analyzed the critical amino acids’ conservation across 12 tick species, utilizing amino acid homology comparisons. Molecular docking results showed an affinity energy of -5.6 kcal/mol between terpinolene and the CYP3A8 protein. The interaction between terpinolene and CYP3A8 protein is specifically in the formation of hydrophobic interactions between terpinolene and amino acid residues ARG-103, ARG-104, LEU-106, PHE-109, and ILE-119 (Figure 6). These amino acid residues that interact with terpinolene are all non-conserved amino acids except for ARG-103.

## 3. Discussion

Previous research has demonstrated that terpinolene exhibits significant repellent properties against ticks [30], and the results of the nymphicidal activity test in this study showed that terpinolene has good killing activity against *H. asiaticum*. However, there remains a dearth of specific research on CYP450 family biological properties, such as functional and expression profiles, among ticks. Given the extensive diversity of CYP450 in terms of sequence, function, and substrates, it is complex to ascertain its specific functions. An exploration of the expression patterns of CYP450 across various organs and developmental stages could offer insights into its distinct roles [34]. Therefore, by way of this study, we are the first to identify, functionally annotate, and analyze the expression pattern of CYP450 genes in *H. asiaticum* under terpinolene stress through genome-associated transcriptomics. Simultaneously, we investigated the molecular mechanisms and tissue distribution characteristics of the differentially highly expressed CYP450 genes in unfed *H. asiaticum* involved in terpinolene metabolism via molecular docking experiments.

Using a genome-associated transcriptome approach, we identified a total of 120 CYP450 genes (35 of which are newly discovered) that contain the complete CYP450 domain in the unfed stage of *H. asiaticum*. Among these, three genes (KAH6930275.1, KAH6930969.1, and MSTRG.28517) were split into two via a homologous correction process. Comparison with other acari species disclosed variances in the number of CYP450 genes among different tick species [10,35]. To elaborate on the role of CYP450 genes in *H. asiaticum*, we analyzed their physicochemical properties. Findings revealed that these genes encoded proteins that can be characterized as unstable, hydrophobic, high-molecular-weight proteins with no signal peptide and 1–3 asymmetrical transmembrane regions. The phylogenetic analysis found the CYP450 genes in *H. asiaticum* to be consistent with the typical classification of insect CYP450 gene families [9], and no genes classified under the new classification, CYP20, were discovered [10]. Notably, the abundance of genes within different *H. asiaticum* CYP450 clans differed significantly, with clan3 being the most populous. Within the insect cohort, clan3 and clan4 have higher abundance compared to clan2 and clan mito [36], but in *H.asiaticum*, CYP450 clan2 (39) showcases significant prevalence compared to clan4 (21). Apart from its engagement in ecdysteroid metabolism and ecdysone biosynthesis [10], CYP2 also contributes to chemical insecticide resistance [37], antioxidant activity, and immune responses [38]. These multifactorial roles and the numerical dominance of clan2 potentially augment its members’ importance and complexity in the developing of *H. asiaticum*. Conserved motif analysis indicates that all 120 *H. asiaticum* CYP450 genes share five identical conserved motifs with insect CYP450s [39,40], implying a functional similarity between tick and insect CYP450 genes.

It is widely acknowledged that gene expression underpins the synthesis of effector proteins necessary to execute biological functions. The expression patterns of CYP450 genes have been identified to be linked with the developmental stage and gender of insects [41,42,43]. Insight into the expression profiles of CYP450 genes in unfed *H.asiaticum* from this study reveals that the seven most prominently expressed genes primarily indicate clans3 (7) and clans2 (2), with the forty-one moderately expressed genes shared among clans3 (22), clans2 (14), and clans4 (5). In contrast, the reduced expression level of CYP450 is associated with 70 additional genes, including clan mito (4). The non-diminished-expression CYP450 genes harbored in unfed *H. asiaticum* could significantly contribute to the organism’s growth and maturation. Indeed, most genes with reduced CYP450 expression in *H. asiaticum* are equally intriguing as they may be activated under specialized conditions [44,45].

The GO enrichment analyses conducted in this research suggest that the functions of the CYP450 in *H. asiaticum* are comparable to those found in *Amrasca biguttula* and *Bactrocera oleae* [46,47]. In particular, GO: 0098754 denotes that CYP450 contributes to detoxification processes in the organism, highlighting the significantly up-regulated gene CYP3A8 (KAH6930993.1) under terpinolene stress. The primary class of the cellular component indicates that CYP450 is a fundamental constituent of *H. asiaticum* cellular composition. Molecular function is dominated by catalytic activity, implying that CYP450 serves as an enzyme to catalyze biochemical reactions.

Based on the KEGG pathway enrichment results, it is clear that terpinolene affects the overall metabolic levels of *H. asiaticum*. The second pathway enriched was steroid hormone biosynthesis (ko00140). This pathway performs similar functions in vertebrates and insects [48]. Steroid hormones in *Drosophila* can have the ability to regulate cell death, metamorphosis, signaling, and apoptosis before programmed cell death [49]. The CYP450 gene is also a significant member of the steroid hormone biosynthesis pathway [50]. In the present study, several CYP450 members of the CYP450 clan2 subfamily were clustered in the steroid hormone biosynthesis pathway, including the CYP3A8 gene from clan3. Expression data for these genes show that their expression levels are at an abnormal level, suggesting that the acute toxicity mechanism of terpinolene against unfed nymph *H. asiaticum* may act mainly by affecting the biosynthesis of steroid hormones leading to signaling abnormality and regulating cell death. In contrast, the chronic toxicity may be accomplished by involving the metamorphosis of the ticks. The CYP3A8 gene was typically expressed in mRNA in the control group and showed highly significant up-regulation of expression after terpinolene treatment. This suggests that the protein encoded by the CYP3A8 gene plays a vital role in terpinolene metabolism, and also indicates that the CYP450 gene family has an important and complex function in the life of *H. asiaticum*. This study’s results differ from the mechanism by which monoterpenoids regulate voltage-gated Ca^2+^ channels and affect “lifetime regulation”, etc. [51], indicating that ticks have different metabolic mechanisms for toxic monoterpenoids.

The screening of key CYP450 family members for metabolizing terpinolene in the present study was inspired by the fact that the cause of resistance shown by pests to insecticides is related to the overexpression of CYP450 genes [18,52,53]. The metabolism of monoterpenes in insects is correlated with genes from clan3 and clan4 [54,55], and, as our study suggests, the metabolism of monoterpenes in *H. asiaticum* also comes from the clan3 (CYP3A8). Considering the differences in organizational expression profiles of the CYP450 genes [56,57,58], we proceeded to perform a qRT-PCR on the CYP3A8 gene of *H. asiaticum*. A closer examination of the tissue distribution of the CYP3A8 gene in *H. asiaticum* revealed it to be tissue-specific, predominantly found in the fat body, in accordance with previous studies indicating that CYP3 genes located in fat bodies are linked with abamectin tolerance [59]. These findings suggest that the fat body is the primary site for CYP3 gene expression.

Molecular docking serves as a computer-aided drug design method and is noted to facilitate the production of acaricides that exhibit superior resistance effects [4]. It also plays a pivotal role in understanding molecular interactions in various biological processes, including signal transduction, enzyme catalysis, and drug action [60]. Currently, molecular docking is leveraged to assess the binding capacity and mechanism of natural products to tick receptor targets or detoxification enzymes [61,62,63]. In this study, we evaluated the binding mechanism of *H. asiaticum* CYP3A8 protein to terpinolene by using molecular docking. Results indicated that the CYP3A8 protein from *H. asiaticum* hydrophobically interacted with terpinolene via the amino acid residues ARG-103, ARG-104, LEU-106, PHE-109, and ILE-119, exhibiting strong binding capacity (affinity = −5.6 kcal/mol). Molecular docking facilitates the exploration of receptor binding sites and explains the varying potencies of compounds [64]. Analysis of the conservation of the five amino acid residues from *H. asiaticum*, which bind to terpinolene, in the homologous sequences of the 12 species suggested that four are non-conserved sites, with the exception of ARG-103. Thus, different tick species and CYP450 genes in *H. asiaticum* may have varying metabolism potencies for terpinolene. These findings provide novel insights for further investigation into the mechanism of terpene metabolism in ticks.

## 4. Materials and Methods

### 4.1. Ticks

*H. asiaticum* was cultivated using previous laboratory culture methods [65]. Briefly, 2-week-old unfed larvae were selected for inoculation into the outer ear part of New Zealand rabbits with adhered ear covers. After about 5 days, the naturally falling engorged larvae were collected. They were placed to molt at 27 ± 2 °C and 80 + 5% relative humidity. During this period, they were not touched by any chemical acaricides.

### 4.2. Chemical Reagents and Nymph Immersion Test

Terpinolene (>95%) was purchased from Beijing Bailing Wei Technology Co. (Beijing, China), while Amitraz was sourced from Fengcheng Animal Medicine Co. (Fengcheng, China). The Nymph Immersion Test (NIT), described by Desmond O. Agwunobi et al. [51], was employed with slight adjustments. Specifically, terpinolene was dissolved in a 70% ethanol solution to make working solutions with concentrations of 2.5 mg/mL, 5 mg/mL, 10 mg/mL, and 20 mg/mL. A 70% ethanol solution served as a negative control and the amitraz solution as a positive control. Three biological replicates of the same concentration of reagent or control were set up in the immersion tests, with each replicate containing 20 ticks, and each immersion was completed for 5 min. Five min later, the ticks were dried of the drug reagent on filter paper and placed under laboratory conditions for 24 h. After this duration, the ticks were gently compressed using tweezers under a stereomicroscope, and any ticks failing to exhibit signs of movement were considered dead. The LC_50_ and its associated 95% confidence intervals (95% CI) were calculated using probit analysis in SPSS v20.0.

### 4.3. MFO Activity Assay

Based on NIT results, approximately 400 tick samples were selected as MFO (mixed function oxygenase, MFO) enzyme activity assay tick samples under LC_20_, LC_50_, and LC_80_ treatment conditions, using a 70% ethanol solution as a control. Three biological replicates were performed for each treatment concentration and four technical replicates were performed in each biological replicate.

### 4.4. Transcription Sequencing Sample Preparation

The LC_50_ of unfed nymphs of *H. asiaticum* under terpinolene stress was used as the experimental group, while 70% ethanol solution was used as the control group. Each treatment set was replicated ten times. After a 24 h treatment period, surviving ticks were isolated and segregated into clusters containing approximately 20 mg of ticks. Five such groups were prepared for both the treatment and control samples. After that, the ticks were cleansed with 200 µL of pre-cooled 70% ethanol solution and then with pre-cooled PBS solution. Next, they were dried using clean filter paper. All gathered samples were pat-dried utilizing clean filter paper. To conclude, tick samples were quickly frozen in liquid nitrogen for half an hour and subsequently stored at −80 °C.

### 4.5. RNA Extraction, Library Construction, and Sequencing

Total RNA was extracted with a Trizol reagent kit (Invitrogen, Carlsbad, CA, USA) following the manufacturer’s protocol. The RNA quality was evaluated on an Agilent 2100 Bio-analyzer (Agilent Technologies, Palo Alto, CA, USA) and verified with RNase-free agarose gel electrophoresis. After extraction of total RNA, eukaryotic mRNA was enriched with Oligo(dT) beads, while prokaryotic mRNA was enriched through rRNA removal using the Ribo-Zero™ Magnetic Kit (Epicentre, Madison, WI, USA). Subsequently, the enriched mRNA was fragmented into short segments utilizing fragmentation buffer and reverse-transcribed into cDNA using random primers. The second-strand cDNA was synthesized using DNA polymerase I, RNase H, dNTP, and buffer. Following this, the cDNA fragments were purified with a QiaQuick PCR extraction kit (Qiagen, Venlo, The Netherlands), repaired at the ends, added to A base, and ligated to Illumina sequencing adapters. The size of the ligation products was chosen by agarose gel electrophoresis, PCR-amplified, and sequenced using the Illumina Novaseq 6000 by Gene De Novo Biotechnology Co. (Guangzhou, China).

### 4.6. Cleaning, Alignment, Quantification, and Profiling

Fastp (version 0.18.0) [66] was employed for read filtration using the following parameters: (i) adaptor-containing reads were removed; (ii) reads with over 10% unknown nucleotides (N) were discarded; and (iii) low-quality reads having more than 50% bases with a *Q*-value ≤ 20 were eliminated. An index of the *H. asiaticum* reference genome (GCA_013339685.2) was constructed, and then the mapping of the paired-end clean reads onto the reference genome using HISAT2 v2.4 [67] was conducted, with other parameters set to default. Each sample’s mapped reads were assembled by StringTie v1.3.1 [68,69], employing a reference-based approach. Novel genes are defined as those identified in the current sequencing results but not encompassed in the reference genome or in the collection of reference genes, post-reconstruction of the transcripts through Stringtie. Transcripts Per Kilobase of exon model per Million mapped reads (TPM) values were tabulated for each transcription region, quantifying its expression abundance and variations with the assistance of RSEM [70].

### 4.7. qRT-PCR Validation of Transcriptome Data

Three up-regulated and three down-regulated genes were randomly chosen from the transcriptomic gene expression results. The verification of the changes in gene expression was performed through qRT-PCR. Details about the six randomly selected gene-specific primers and specific experimental steps can be found in Tables S5 and S6 of File S1. The internal reference gene EIF1α was applied [71], and the expression levels of these genes were analyzed using the 2^−ΔΔCT^ method.

### 4.8. Identification of Hyalomma Asiaticum CYP450s

The CYP450s of *Drosophila melanogaster*, *Aedes aegypti*, *Parasteatoda tepidariorum*, and *Centruroides sculpturatus* were retrieved from the NCBI database and used as query sequences. This sequence was used to identify CYP450 candidates from *H. asiaticum* genomic data and to retrieve CYP450 candidates via blast+ (*E* < 0.05). Concurrently, the hmmer method was also utilized to search for CYP450 candidates in *H. asiaticum* by using the CYP450 HMM file (PF00067, *E* < 0.05). These gene sequences were then refined using Fgenesh+ (http://www.softberry.com/, accessed on 25th April 2024). Ultimately, the outcomes from both methods were combined in the NCBI CDD database for domain identification, and genes that contained complete domains and exhibited Helix–C, Helix–I, Helix–K, PERF, and heme-binding classical motifs were identified as final *H. asiaticum* CYP450 candidates. The protein physicochemical property analysis was conducted through the ExPASy online website (accessed on 7th June 2024).

### 4.9. Phylogenetic Analysis

The CYP450s amino acid sequences for *Aedes aegypti*, *Anopheles gambiae*, *Drosophila melanogaster*, and *Tetranychus urticae* were retrieved from the NCBI database (accessed on 6th June 2024). At the same time, those for *Ixodes Scapularis* CYP450s were obtained as described by Dermauw W. et al. [10]. All sequences underwent multiple sequence comparisons through MAFFT v4.748 [72], trimming via TrimAl v1.2 [73], phylogenetic tree construction via iqtree v2.2.2.6 [74], and results visualization by iTOL (accessed on 15th June 2024) [75].

### 4.10. Screening of Differentially Expressed Genes

The DESeq2 v1.20.0 software was employed to identify differentially expressed genes between the control and experimental groups, using parameters of |log2FC| > 1 and *p* < 0.05. Genes were categorized based on their expressions: low expression for TPM = 1–10, medium expression for TPM = 10–100, and high expression for TPM > 100.

### 4.11. Functional Annotation of CYP450

GO and KEGG annotations were performed using eggNOG–MAPPER and Kofam–KOALA on candidate genes of the CYP450 in *H. asiaticum*. Differential analysis of GO and KEGG pathway were performed using the results of genomic GO and KEGG annotation as background genes.

### 4.12. Molecular Docking

The complete CDS of the *H. asiaticum* CYP3A8 gene, which was significantly up-regulated and expressed, was amplified using PCR. The reaction system included the following: 12.5 μL of 2×Tag PCR Master Mix, 1 μL of F/R primer, 1 μL of cDNA, and 25 μL of ddH_2_O. The PCR conditions were set as follows: pre-denaturation at 95 °C for 5 min; denaturation at 95 °C for 30 s; annealing at 65 °C for 30 s; extension at 72 °C for 30 s for 30 cycles; a final extension at 72 °C for 5 min; and termination at 4 °C (primer information is in Table S5 of File S1). The obtained *H. asiaticum* CYP3A8 nucleotide sequence was translated into amino acids by ORF Finder (accessed 26th June 2024) and then homology was modeled in the SWISS-MODEL server (accessed 29th September 2024) using the human CYP450 3A5 protein (PDB ID: 7LAD) from the PDB database (accessed 29th September 2024) as a template. Blind molecular docking using the CYP3A8 protein model as a receptor and the terpinolene molecular structure (CAS: 586–62–9) as a ligand was conducted in Autodock Vina v1.2 and visualization was conducted by pymol v 2.5.0. Conservation analysis of amino acid residues of the *H. asiaticum* CYP3A8 protein that interact with terpinolene among homologous proteins of 11 tick species (*Rhipicephalus sanguineus*, *R*. *microplus*, *Amblyomma americanum*, *Dermacentor silvarum*, *Ixodes hexagonus*, *I. scapularis*, *I. pacificus*, *D. andersoni*, *D. albipictus*, *I. persulcatus*, and *Ornithodoros turicata*) was accomplished by Jalview v2.11.4.0 software.

### 4.13. Tissue Distribution of CYP450 Genes of Hyalomma Asiaticum Involved in Terpinolene Metabolism

Three naturally shed engorged female ticks of pure blood were selected, and their malpighian tubule, midgut, ovary, and fat body were independently isolated in a 1xPBS solution. Quantifying expression in these various tissues parallels the information presented in the qRT-PCR validation section.

## 5. Conclusions

This research suggests that terpinolene demonstrates a high efficacy in eliminating unfed nymph *H. asiaticum*. The transcriptome-associated genome data of terpinolene stress yielded 120 *H. asiaticum* CYP450 genes, including data of the entire CYP450 domain and five conserved motifs. These genes’ biological properties were comprehensively evaluated by scrutinizing their genetic evolution, expression patterns, and functions. The distribution, binding characteristics, and preliminary investigation of essential CYP450 genes, implicated in terpinolene metabolism in *H. asiaticum*, were obtained via molecular docking and qRT-PCR. Although the classification of *H. asiaticum* CYP450 genes mirrored that of insects, the expression level varies. Functional enrichment results indicated that the CYP450 genes are present in several critical physiological systems of *H. asiaticum* and play different biological roles. The mechanism of the toxic effect of terpinolene on *H. asiaticum* may be to cause an abnormality in the steroid hormone biosynthesis pathway. CYP3A8, significantly overexpressed in the differential gene set and crucial for metabolizing terpinolene, appears mainly in the fat body tissue. Molecular docking outcomes revealed that five critical amino acids in CYP3A8 engage in a hydrophobic interaction with terpinolene. Analysis of these five key amino acids’ conservation across different tick species leads to the proposition that metabolic potencies for terpinolene might differ among tick species. These findings provide valuable insights into the biological function of the CYP450 gene and the metabolic mechanism of metabolizing natural products in *H. asiaticum*.

## Data Availability

The original contributions presented in the study are included in the supplementary material, further inquiries can be directed to the corresponding authors.

## Supplementary Materials

The following supporting information can be downloaded at https://www.mdpi.com/article/10.3390/ijms252111467/s1.
