# Supplementary material for: Biological Characteristics of the Cytochrome P 450 Family and the Mechanism of Terpinolene Metabolism in Hyalomma asiaticum (Acari: Ixodidae)
Source: Int J Mol Sci. 2024 Oct 25;25(21):11467. doi: 10.3390/ijms252111467 (PMC11546871; doi:10.3390/ijms252111467)
Supplement: Supplementary file 1 [file ijms-25-11467-s001.zip › ijms-3193866-supplementary material.pdf]

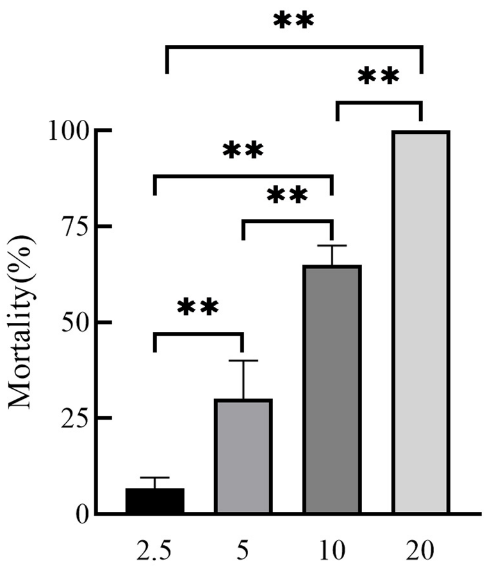

Figure S1. Toxic effects of different concentrations of terpinolene against unfed nymph *H. asiaticum.* ** indicates a comparative analysis of differences between groups ($p < 0.01$)

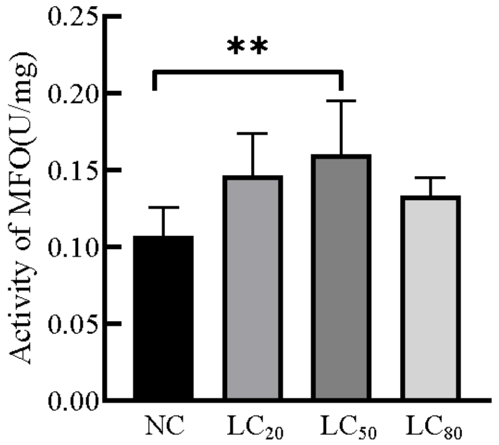

Figure S2. Effects of different concentrations of terpinolene on the MFO enzyme activity of unfed nymph *H. asiaticum.* ** indicates a comparative analysis of differences between groups ($p < 0.01$)

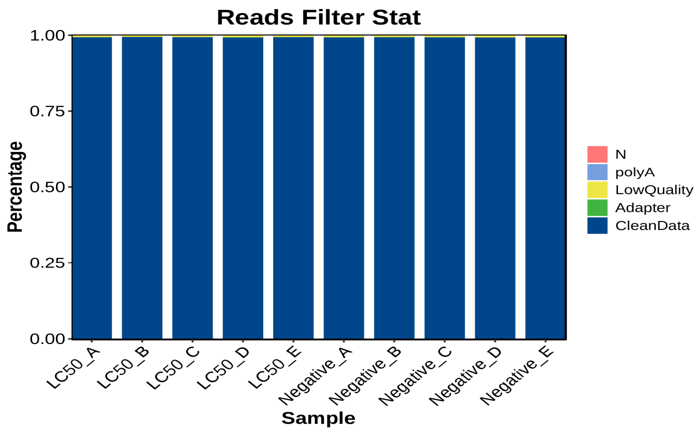

Figure S3. Distribution of data pre-processing (percent)

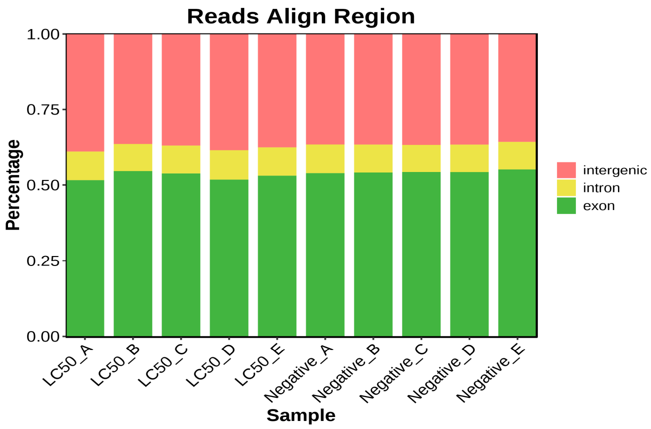

Figure S4. Comparison of statistics for the reference region

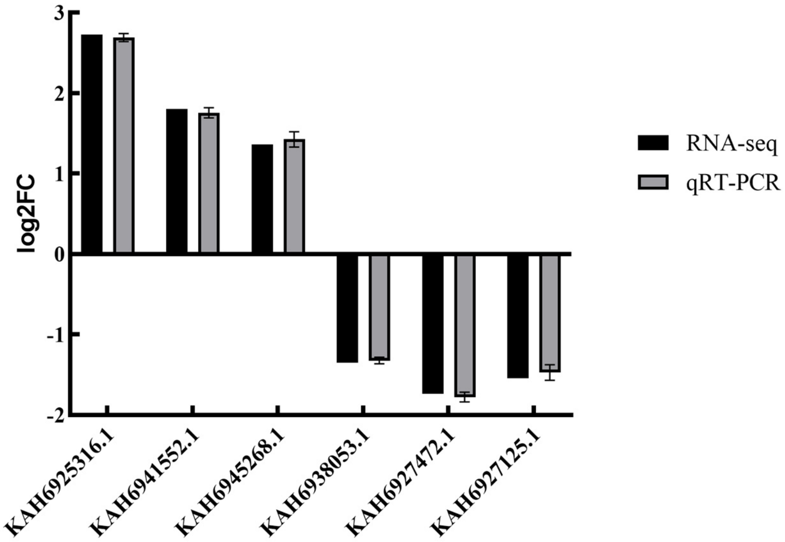

Figure S5. Results of qRT-PCR validation of 6 selected genes

Table S1. Statistical table of base information

Sample	RawData(bp)	CleanData(bp)	After filter_Q20(%)	After filter_N(%)	After filter_GC(%)
LC50_A	6706065000	6635016550	6469321917 (97.50%)	107740 (0.00%)	3183081729 (47.97%)
LC50_B	5503898700	5452916291	5334669683 (97.83%)	28398 (0.00%)	2725408588 (49.98%)
LC50_C	6543219300	6477552045	6324136155 (97.63%)	83810 (0.00%)	3222993969 (49.76%)
LC50_D	6480355200	6395900411	6246340407 (97.66%)	80615 (0.00%)	3095595100 (48.40%)
LC50_E	5595771000	5537384248	5421364829 (97.90%)	26573 (0.00%)	2723301944 (49.18%)
Negative_A	6596013000	6529727713	6398432104 (97.99%)	122243 (0.00%)	3231532319 (49.49%)
Negative_B	6624147300	6556251107	6419971419 (97.92%)	103740 (0.00%)	3233543677 (49.32%)
Negative_C	6786691500	6714081282	6563573660 (97.76%)	141370 (0.00%)	3346856190 (49.85%)
Negative_D	6430086300	6366167691	6209157176 (97.53%)	107013 (0.00%)	3154394228 (49.55%)
Negative_E	6869149200	6802547223	6611707883 (97.19%)	137014 (0.00%)	3383879503 (49.74%)

Table S2. Comparison of ribosome statistics

Sample	clean_reads	Mapped_Reads(%)	Unmapped_Reads(%)
LC50_A	44407602	2042406 (4.60%)	42365196 (95.40%)
LC50_B	36478854	454140 (1.24%)	36024714 (98.76%)
LC50_C	43330286	523460 (1.21%)	42806826 (98.79%)
LC50_D	42871658	1470440 (3.43%)	41401218 (96.57%)
LC50_E	37067356	816654 (2.20%)	36250702 (97.80%)
Negative_A	43657414	604586 (1.38%)	43052828 (98.62%)
Negative_B	43862746	1122356 (2.56%)	42740390 (97.44%)
Negative_C	44928336	670362 (1.49%)	44257974 (98.51%)
Negative_D	42544102	527472 (1.24%)	42016630 (98.76%)
Negative_E	45462458	613202 (1.35%)	44849256 (98.65%)

Note: Sample:sample name; clean_reads: number of high quality reads; Mapped_Reads (%): number of reads that match the ribosomes of the species and the percentage (based on cleand reads) of those that do. Unmapped_Reads (%): number of reads that do not match the ribosome and percentage (based on cleand reads).

Table S3. Comparison of reference genome statistics

Sample	Total	Unmapped(%)	Unique_Mapped(%)	Multiple_Mapped(%)	Total_Mapped(%)
LC50_A	42365196	5594909 (13.21%)	35906417 (84.75%)	863870 (2.04%)	36770287 (86.79%)
LC50_B	36024714	4603938 (12.78%)	30709244 (85.24%)	711532 (1.98%)	31420776 (87.22%)
LC50_C	42806826	5338603 (12.47%)	36595082 (85.49%)	873141 (2.04%)	37468223 (87.53%)
LC50_D	41401218	5234387 (12.64%)	35304531 (85.27%)	862300 (2.08%)	36166831 (87.36%)
LC50_E	36250702	4555750 (12.57%)	30951065 (85.38%)	743887 (2.05%)	31694952 (87.43%)
Negative_A	43052828	5368134 (12.47%)	36773988 (85.42%)	910706 (2.12%)	37684694 (87.53%)
Negative_B	42740390	5315049 (12.44%)	36546748 (85.51%)	878593 (2.06%)	37425341 (87.56%)
Negative_C	44257974	5435722 (12.28%)	37918676 (85.68%)	903576 (2.04%)	38822252 (87.72%)
Negative_D	42016630	5374005 (12.79%)	35785111 (85.17%)	857514 (2.04%)	36642625 (87.21%)
Negative_E	44849256	5780402 (12.89%)	38155585 (85.08%)	913269 (2.04%)	39068854 (87.11%)

Note: Sample: Sample name; Total: the number of reads after filtering ribosomes, known as valid reads; Unmapped (%): the number of reads that are not matched to the reference genome and the percentage of valid reads; Unique_Mapped (%): the number of reads uniquely matched to the reference genome and the percentage of valid reads; Multiple_Mapped (%): the number of reads and the percentage of valid reads on the reference genome for multiple matching; Total_Mapped (%) :The number of reads that can be localised to the genome and the percentage of valid read.

Table S4. Statistics on genetic testing

sample	Refer_Genes	sequenced_Refer_Genes(%)	Novel_Genes	sequenced_Novel_Genes(%)	Total_Genes	sequenced_Total_Genes(%)
all	29657	16210 (54.66%)	4891	4891 (100.00%)	34548	21101 (61.08%)
LC50_A	29657	11376 (38.36%)	4891	4466 (91.31%)	34548	15842 (45.86%)
LC50_B	29657	11526 (38.86%)	4891	4446 (90.90%)	34548	15972 (46.23%)
LC50_C	29657	11674 (39.36%)	4891	4500 (92.01%)	34548	16174 (46.82%)
LC50_D	29657	11508 (38.80%)	4891	4485 (91.70%)	34548	15993 (46.29%)

LC50_E	29657	11571 (39.02%)	4891	4502 (92.05%)	34548	16073 (46.52%)

Continuation of table S4

sample	Refer_Genes	sequenced_Refer_Genes(%)	Novel_Genes	sequenced_Novel_Genes(%)	Total_Genes	sequenced_Total_Genes(%)
Negative_A	29657	11875 (40.04%)	4891	4479 (91.58%)	34548	16354 (47.34%)
Negative_B	29657	11922 (40.20%)	4891	4473 (91.45%)	34548	16395 (47.46%)
Negative_C	29657	11637 (39.24%)	4891	4446 (90.90%)	34548	16083 (46.55%)
Negative_D	29657	11328 (38.20%)	4891	4422 (90.41%)	34548	15750 (45.59%)
Negative_E	29657	11562 (38.99%)	4891	4432 (90.62%)	34548	15994 (46.30%)

Note: Refer_Genes: the total number of genes in the reference gene set (or the set of reference genes); sequenced_Refer_Genes(%): the total number of Refer_Genes detected by the sequencing result and the percentage of Refer_Genes; Novel_Genes: the number of novel genes detected by the project; sequenced_Novel_Genes(%): the total number of Novel_Genes detected by sequencing results and the percentage of Novel_Genes; Total_Genes: total number of all genes, including reference genome and new genes; sequenced_Total_Genes(%): total number of genes detected by sequencing results and percentage of all_Genes.

Table S5. Information on qRT-PCR primers for transcriptome data validation

	NCBI accession	Gene Names	Forward Primer(5´-3´)	Reverse Primer(5´-3´)
Up-regulated	KAH6925316.1	Epoxide hydrolase 4 isoform X1	AGCCGAATTCTACAGCGAGA	CAGCCCTTGGTGACGTAATG
	KAH6941552.1	Alpha-crystallin A chain-like	GACAACAGCGTCGTCATTCA	ACGTACCGTTAGGCTTGAGA
	KAH6945268.1	Sulfotransferase	CAAGCAGGATGCCAACAACT	TACAGCATGCCTGAAGGGAA
Down-regulated	KAH6927472.1	Juvenile hormone acid O-methyltransferase-like	GACGCAGGACTGCATTTGAT	GAAGGCGTCCAAGAAGTCAC
	KAH6927125.1		ACGGCTTACGTGCTTTCTTC	TGGCGAAATCGTTTGTCGAA
	KAH6938053.1	Acireductone dioxygenase-like isoform X2	GTGCTGGATGCCCAAAGAAT	CTTCGTACGACGAGGTCTCA
	KAH6930993.1	Cytochrome P 450 3A8	TACGTCCTCGGTGATTCTGG	CATGATGCATGGCGTACACA
Complete cds amplification	KAH6930993.1	Cytochrome P 450 3A8	ATGGAGCTGATTGGTCTA	TCATCGCTCTGGTCTCTC

Table S6. qPCR detection reaction system and reaction conditions

Reagent	Volume(20 μL)	Condition	Step
SYBR qPCR SuperMix	10μL	95 ℃，30 s	Premutability
Forward primer	0.4μL	95℃，5s	45 cycles
Reserve primer	0.4μL	60℃，30s	
cDNA templete	1μL	95 ℃，15 s	
RNase-free water	7.2μL	60℃，60s	Dissolution curve
		95 ℃，15 s	
